# Supplementary material for: A Novel Mouse Model of TGFβ2-Induced Ocular Hypertension Using Lentiviral Gene Delivery
Source: Int J Mol Sci. 2022 Jun 21;23(13):6883. doi: 10.3390/ijms23136883 (PMC9266301; doi:10.3390/ijms23136883)
Supplement: Supplementary file 1 [file ijms-23-06883-s001.zip › ijms-1708383-supplementary.pdf]

Supplementary Materials:

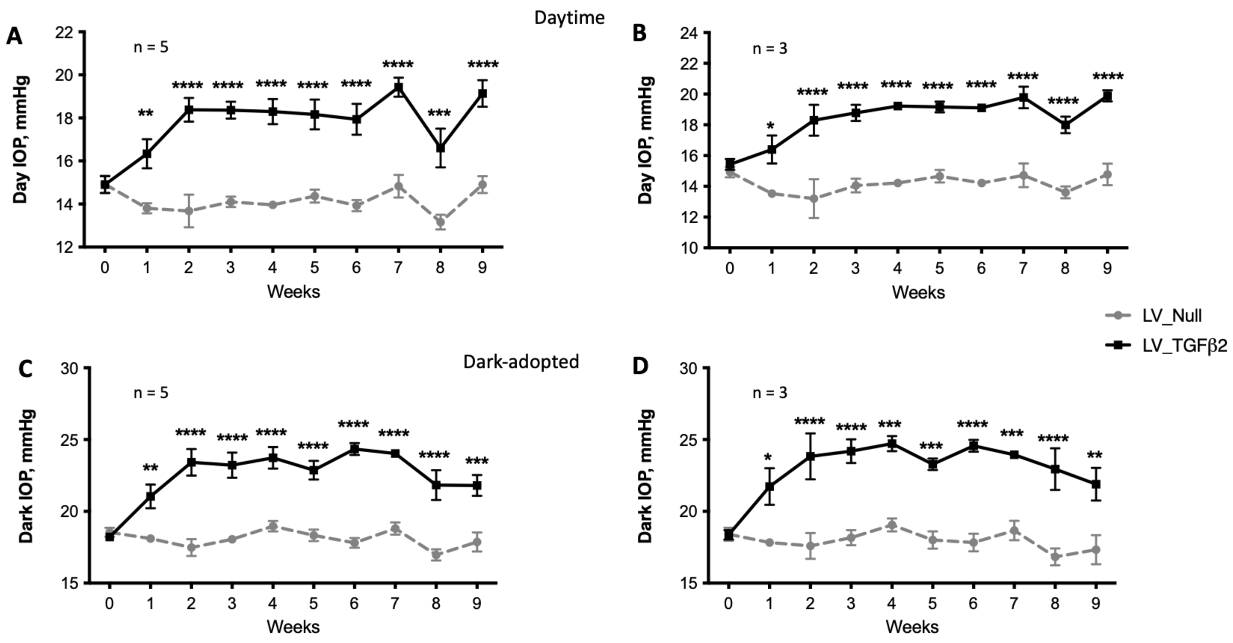

Figure S1. Day and dark IOP measurements under isoflurane influence for LV_TGFβ2 injected C57BL/6J mice. Intravitreal injections of LV_Null (control) and LV_TGFβ2 vectors ($n = 5$ each) were administered in the contralateral eyes of C57BL/6J mice (Males) following baseline IOP measurements. Weekly monitoring of daytime **(A, B)** and dark **(C, D)** IOPs showed significant and sustained IOP elevation 2 weeks post-injections. Mice with LV_TGFβ2-induced IOPs **(B, D)** showing Δ change of >2.5 mmHg was segregated from mice observed with no IOP elevation. Repeated measures two-way ANOVA with Bonferroni post-hoc analysis, data represented as mean ± SEM, $*p < 0.05$, $**p < 0.01$, $***p < 0.001$. (Δ - *delta change).*

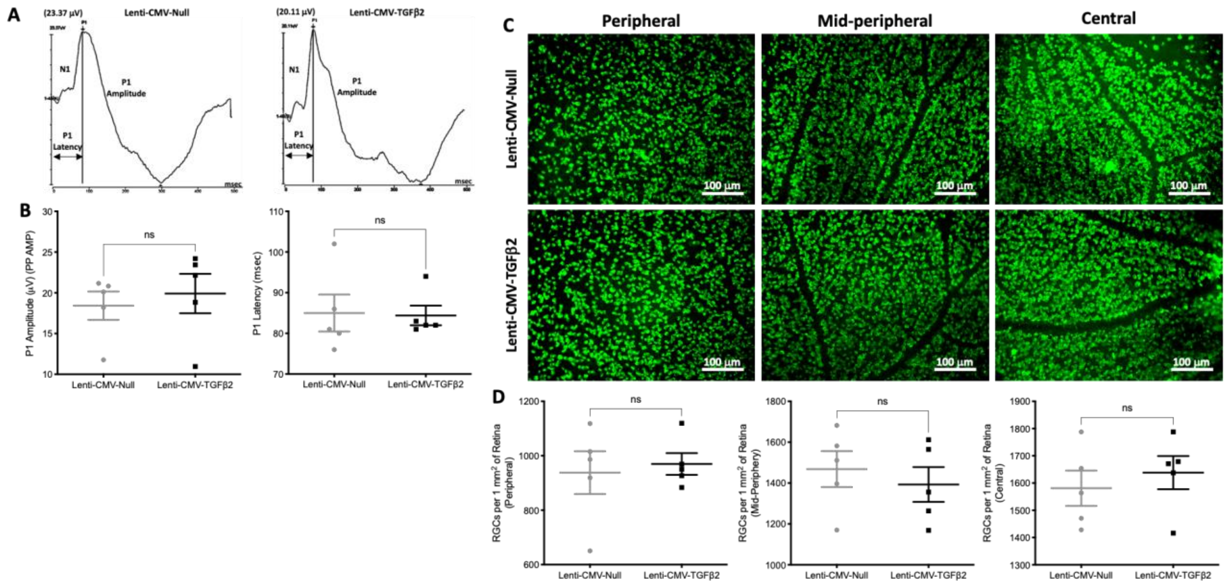

Figure S2. Ocular hypertension induced by LV_TGFβ2 injections did not result in structural and functional loss of RGCs. C57BL/6J mice (*n* = 5) intravitreally injected with LV_Null (control) and LV_TGFβ2 vectors in contralateral eyes were evaluated for changes in RGC functions **(A, B)** via PERG 8 weeks post-injection (refer to Fig S1 for IOP elevation data). **(A)** Representative wave graphs for LV_Null and LV_TGFβ2 injected eyes. **(B)** No significant changes in the amplitude and latencies were observed between the treated eyes, thus demonstrating no functional loss of RGCs. **(C, D)** Later, retinas of these mice were isolated 9 weeks post-injections and were stained with RBPMS to determine changes in RGC count between control and LV_TGFβ2 injected eyes **(C)** Representative images of peripheral, mid-peripheral or central regions of retinas (scale 100 µm). **(D)** No significant changes in RGC counts were observed in any regions of the retinas. Paired (one-tailed) student *t* test, data represented as mean ± SEM.

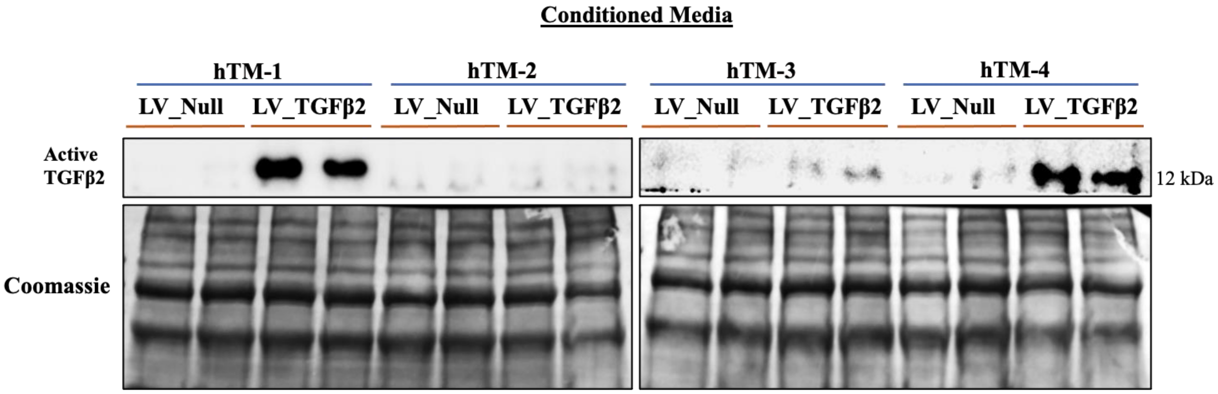

Figure S3. LV_TGFβ2 transduction of primary human TM cells induces active TGFβ2 expression in the conditioned media. Primary human TM cells treated with 5 MOI viral load of LV_Null and LV_TGFβ2 vectors were incubated for 11 days (n = 4). Active TGFβ2 levels were determined in conditioned media of the transduced cells. The total protein loading was determined via Coomassie staining of the PVDF membrane.
